# Clinical characteristics of patients with autoimmune nodopathy with anti-neurofascin155 antibodies

**DOI:** 10.3389/fimmu.2024.1345953

**Published:** 2024-04-25

**Authors:** Jiwei Zhang, Xiaotong Hou, Liting Wei, Jinshun Liu, Shibo Li, Yifan Guo, Hongbo Liu, Yan Jiang

**Affiliations:** ^1^ Department of Neurology, the First Affiliated Hospital of Zhengzhou University, Zhengzhou, China; ^2^ Translational Medicine Center, the First Affiliated Hospital of Zhengzhou University, Zhengzhou, China; ^3^ Department of Neurology, Luoyang Central Hospital, Luoyang Central Hospital Affiliated to Zhengzhou University, Luoyang, China

**Keywords:** chronic inflammatory demyelinating polyradiculoneuropathy, autoimmune nodopathy, nodes of Ranvier, anti-neurofascin155 antibodies, clinical characteristics

## Abstract

**Background:**

According to the latest guidelines on chronic inflammatory demyelinating polyradiculoneuropathy (CIDP), patients with CIDP with anti-neurofascin 155 (NF155) antibodies are referred to as autoimmune nodopathy (AN), an autoimmune disorder distinct from CIDP. We aimed to compare the clinical data of patients with AN with anti-NF155 antibodies with those of anti-NF155 antibodies-negative patients with CIDP, and to summarize the clinical characteristics of patients with AN with anti-NF155 antibodies.

**Methods:**

Nine patients with AN with anti-NF155 antibodies and 28 serologically negative patients with CIDP were included in this study. Diagnosis was made according to the diagnostic criteria in the European Academy of Neurology (EAN)/Peripheral Nerve Society (PNS) guidelines on CIDP published in 2021. Demographics, clinical manifestations, electrophysiological examination, cerebrospinal fluid (CSF) tests, and response to treatment were retrospectively analyzed.

**Results:**

Compared with serologically negative patients with CIDP, those patients with AN with anti-NF155 antibodies were younger (*p*=0.007), had a younger onset age (p=0.009), more frequent ataxia (*p*=0.019), higher CSF protein levels (*p*=0.001), and more frequent axon damage in electrophysiology (*p*=0.025). The main characteristics of patients with AN with anti-NF155 antibodies include younger age and onset age, limb weakness, sensory disturbance, ataxia, multiple motor−sensory peripheral neuropathies with demyelination and axonal damage on electrophysiological examination, markedly elevated CSF protein levels, and varying degrees of response to immunotherapy.

**Conclusions:**

Patients with AN with anti-NF155 antibodies differed from serologically negative patients with CIDP in terms of clinical characteristics. When AN is suspected, testing for antibodies associated with the nodes of Ranvier is essential for early diagnosis and to guide treatment.

## 1 Introduction

Chronic inflammatory demyelinating polyradiculoneuropathy (CIDP) is an autoimmune neuropathy characterized by demyelination of the peripheral nervous system that progresses for more than 8 weeks with a remission−relapse course (1). Typical clinical symptoms include bilateral symmetrical limb weakness with sensory deficits. The disease is most commonly accompanied by albuminocytologic dissociation in cerebrospinal fluid (CSF). Electrophysiological examination shows demyelinating changes, such as slowing of peripheral nerve conduction velocity (CV), prolongation of distal motor latency (DML), conduction block (CB), temporal dispersion (TD), and prolongation of F-wave latency. Patients can benefit from immunotherapy (2).

**Figure 1:**
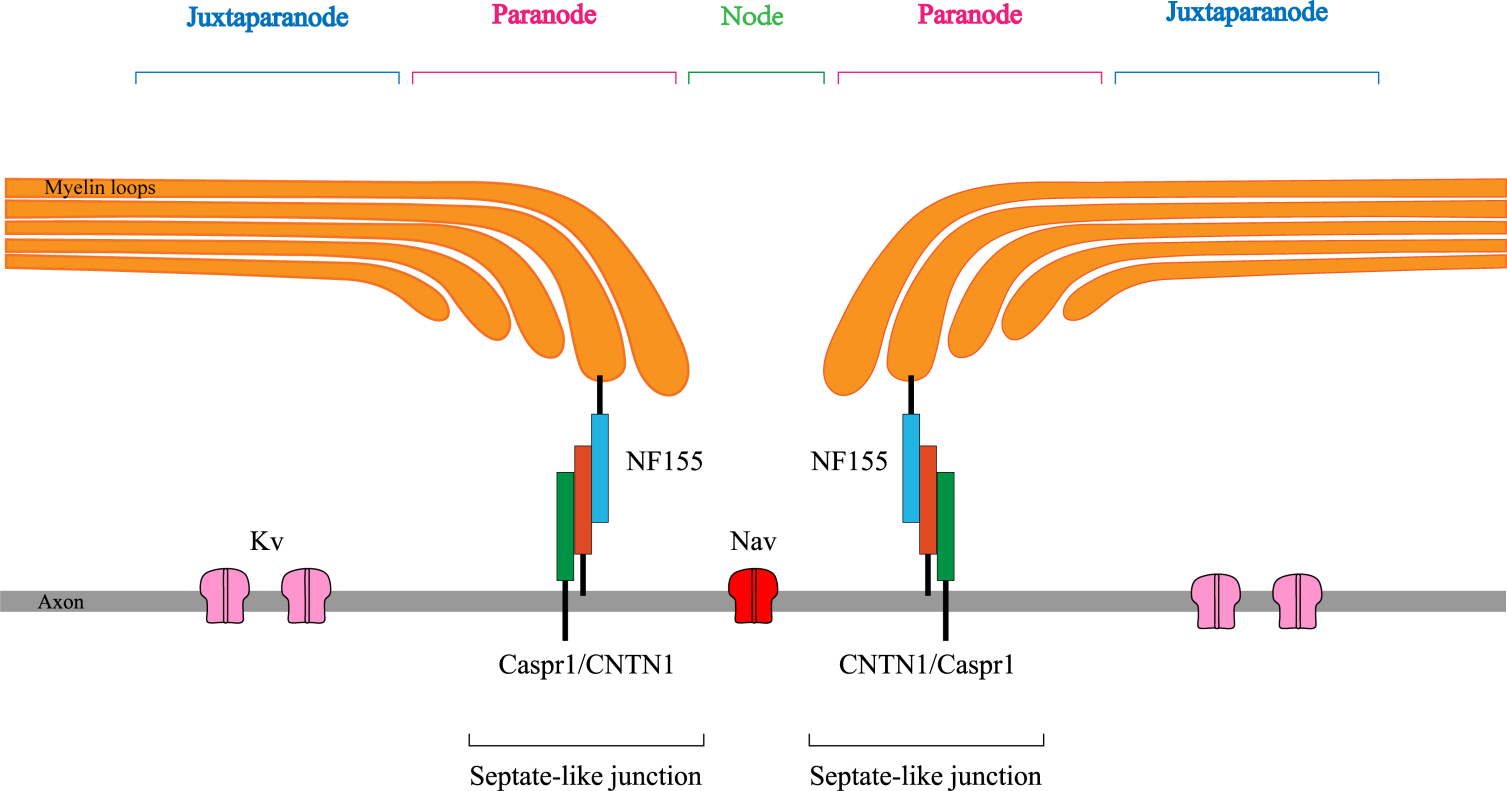
Sketchy anatomical map of the node of Ranvier. NF155, neurofascin155; CNTN1, contactin1; Caspr1, contactin-associated protein1; Kv, Voltage-gated K^+^ channel; Nav, Voltage-gated Na^+^ channel.

In recent years, advances in CIDP research have focused on autoantibodies against the nodal/paranodal protein of nodes of Ranvier (NR) (**Figure 1**). These antibodies are primarily raised against neurofascin 155 (NF155), contactin 1 (CNTN1), or contactin-associated protein 1 (Caspr1) (3). They mediate a unique neuropathy distinct from typical CIDP. In 2021, the most recent guidelines on the diagnosis and treatment of CIDP named this type of neuropathy as autoimmune nodopathy (AN) and recognized it as a relatively separate category of disease entity (2, 4). Pathogenicity has been demonstrated for these antibodies, which have been identified as specific biomarkers for the diagnosis and treatment guidance of AN (5, 6).

Patients with AN have specific clinical characteristics that vary with positive antibodies. The anti-NF155 antibody is the most common nodal/paranodal antibody detected in AN (5). In this study, we compared and analyzed the clinical data of patients with AN with anti-NF155 antibodies and serologically negative patients with CIDP, and summarized the demographics, clinical manifestations, electrophysiological characteristics, CSF tests, and the treatment of patients with AN with anti-NF155 antibodies to improve the clinicians’ understanding of the disease for early recognition, diagnosis, and therapy.

## 2 Methods

### 2.1 Patients selection

Clinical data were retrospectively collected from 37 patients with CIDP who were diagnosed in the Department of Neurology of the First Affiliated Hospital of Zhengzhou University from August 2019 to August 2023. Nine patients were positive for anti-NF155 antibodies and 28 patients were serologically negative. The inclusion criteria for all patients met the diagnostic criteria for CIDP according to the latest EFNS/PNS 2021 guidelines. We exclude patients with peripheral neuropathies due to other etiologies such as poisoning, trauma, malignant tumors, and diabetes. The following parameters were planned to be assessed during electrophysiological examination: DML, motor conduction velocity (MCV), compound muscle action potential (CMAP) amplitude, F-wave latency of the median, ulnar, peroneal, and tibial nerves, and sensory nerve action potential (SNAP) amplitude and sensory conduction velocity (SCV) of the median, ulnar, superficial peroneal, and sural nerves. The efficacy of treatment was assessed according to change in the Hughes Disability Scale score, i.e., △Hughes (Hughes score at discharge-Hughes score at admission), and the presence or absence of clinical improvement: △Hughes<0 was defined as effective, △Hughes=0 with subjective or objective improvement was defined as partially effective, and △Hughes≥0 without any improvement was defined as ineffective. This study was approved by the Ethics Committee of our hospital.

### 2.2 Cell-based assay

The serum samples of the patients were obtained before treatment and sent to the Neurology Laboratory of the First Affiliated Hospital of Zhengzhou University or Zhengzhou jinyu Clinical Laboratory Center. Fixed CBA was used for antibody detection at both institutes.

The specific steps of the CBA approach include the following: HEK 293 T cells were cultured in DMEM medium containing 10% fetal bovine serum. Cells were transfected with a green fluorescent protein (GFP)-marked expression vector containing cDNA encoding human NF155 NM_ (001160331) in the presence of lipo2000. After 4 hours, the transfection solution was replaced with DMEM medium containing 10% fetal bovine serum and the culture was continued for 24-36 hours. The cells expressing GFP-NF155 were fixed, permeabilized, and blocked before incubation with diluted sera (1:50) in PBS for 2 h at 37°C. The cells were subsequently treated for 45 min with Alexa Fluor 594-goat anti-human IgG Fcγ (1,1000) and rinsed with PBS three times. Finally, they were observed under a fluorescence microscope.

### 2.3 Statistical analyses

Statistical analysis was performed using the SPSS 25.0 software and drawings were performed using Adobe Illustrator 2020 software. Measurement data are presented as the mean ± standard deviation if normally distributed or as the median with interquartile range if not conforming to normal distribution. Enumeration data are presented as frequencies (percentages). According to their normality, measurement data were analyzed using the t-test or Mann−Whitney U-test. Enumeration data were analyzed using the Fisher’s exact test. *P*<0.05 was considered to indicate statistically significant differences.

## 3 Results

The clinical data of patients with AN with anti-NF155 antibodies and serologically negative patients with CIDP are presented in **Table 1**. We noticed a significantly younger age (33.8 ± 16.5 vs 51.3 ± 15.7, *p*=0.007) and onset age in patients with AN with anti-NF155 antibodies than in the serologically negative patients(33.0 ± 16.3 vs 49.8 ± 15.8; *p*=0.009) and the former group presented with ataxia more frequently (88.9% vs 42.9%, respectively; *p*=0.019). No differences were found between the two groups in terms of sex, onset characteristics, or disease course. None of the nine patients with anti-NF155 antibodies experienced pain. The frequencies of weakness (*p*=0.444), numbness (*p*=0.377), tremor (*p*=0.244), sensory deficiency (*p*=0.216), pain (*p*=0.568), and cranial nerve involvement (p=0.543) were not significantly different between the two groups.

**Table 1:** Comparison of clinical characteristics of patients with AN with anti-NF155 antibodies with serologically negative patients with CIDP.

Characteristics	Anti-NF155	Negative	*p* value
	(n=9)	(n=28)	
Demographics			
Female/Male	1/8	12/16	0.087
**Age (years)**	33.8 ± 16.5	51.3 ± 15.7	**0.007**
**Onset age (years)**	33.0 ± 16.3	49.8 ± 15.8	**0.009**
Onset characteristics, n/N(%)			
Triggering(infection/vaccination)	3/9 (33.3%)	5/28 (17.9%)	0.292
Acute/subacute	3/9 (33.3%)	7/28 (25%)	0.463
Chronic	6/9 (66.7%)	21/28 (75%)	0.463
Course of disease, n/N(%)			
Remitting-relapsing	2/9 (22.2%)	14/28 (50%)	0.141
Progressing	7/9 (77.8%)	14/28 (50%)	0.141
Disease duration (month)	7.0 (5.0, 10.5)	7.5 (3.0, 21.0)	0.931
Clinical manifestations, n/N(%)			
Limb weakness	8/9 (88.9%)	22/28 (78.6%)	0.444
Limb numbness	6/9 (66.7%)	22/28 (78.6%)	0.377
Tremor	2/9 (22.2%)	1/28 (7.1%)	0.244
Sensory deficiency	8/9 (88.9%)	19/28 (67.9%)	0.216
**Ataxia**	8/9 (88.9%)	12/28 (42.9%)	**0.019**
Pain	0/9 (0%)	2/28 (7.1%)	0.568
Cranial nerve involvement	1/9 (11.1%)	5/28 (17.9%)	0.543
Electrophysiologic examination, n/N(%)			
Demyelinating predominant	8/9 (88.9%)	22/28 (78.6%)	0.444
Axon damage predominant	1/9 (11.1%)	6/28 (21.4%)	0.444
**Axon damage**	9/9 (100%)	17/28 (60.7%)	**0.025**
Prolonged DML	9/9 (100%)	24/28 (85.7%)	0.310
Prolonged F-wave latency	4/9 (44.4%)	11/28 (39.3%)	0.541
Reduced CV	9/9 (100%)	28/28 (100%)	NA
CB	1/9 (11.1%)	2/28 (7.1%)	0.578
TD	1/9 (11.1%)	2/28 (7.1%)	0.578
**Reduced CMAP amplitude**	9/9 (100%)	17/28 (60.7%)	**0.025**
CSF examination			
**CSF protein (g/L)**	3.37 (1.71,3.47)	0.79 (0.56,1.64)	**0.001**
Leucocyte (<5/µl)	4.0 (2.0, 7.0)	2.0 (2.0, 4.0)	0.566
Protein cell separation	8/9 (88.9%)	18/28 (64.3%)	0.163
Improvement by treatment, n/N(%)			
Corticosteroids	6/6 (100%)	18/20 (90%)	0.585
IVIG	0/1 (0%)	10/11 (90.9%)	0.167
PE	3/3 (100%)	1/1 (100%)	NA
RTX	1/1 (100%)	0 (0%)	NA
Cyclophosphamide	1/1 (100%)	1/1 (100%)	NA

DML, distal motor latency; CV, conduction velocity; CB, conduction block; TD, temporal dispersion; CMAP, compound muscle action potential; CSF, cerebrospinal fluid; IVIg, intravenous immunoglobulins; PE, plasma exchange; RTX, Rituximab; NA, not applicable. Bolded values represent statistically significant differences in clinical data between the two groups of patients with autoimmune nodopathy with anti-NF155 antibodies and serologically negative patients.

All nine patients with AN with anti-NF155 antibodies exhibited multiple motor−sensory peripheral nerve neuropathy with myelin and axonal involvement (**Supplementary Table 1**). Axonal damage was more frequently seen in patients with AN with anti- NF155 antibodies than in serologically negative patients (100% vs 60.7%, respectively; *p*=0.025).

Most patients with CIDP exhibited elevated CSF protein levels. The mean CSF protein levels were significantly higher in patients with AN with anti-NF155 antibodies than in serologically negative patients (3.37 [1.71, 3.47] vs 0.79 [0.56, 1.64], respectively; *p*=0.001), but the leukocyte counts were not statistically different. Albuminocytologic dissociation was present in most patients with AN with anti-NF155 antibodies.

There was no statistically significant difference in the efficacy of treatment regimens between the two groups of patients. Most of the serologically negative patients with CIDP were effectively treated with corticosteroids and intravenous immunoglobulin (IVIg) therapy. One patient with AN with anti-NF155 antibodies was treated with IVIg but did not respond to it. The other patients with AN with experienced varying degrees of symptomatic relief after receiving immunotherapies. Multiple treatment regimens were often administered to the same patient (**Table 2**).

**Table 2:** Clinical characteristics of nine patients with AN with anti-NF155 antibodies.

Characteristics	Anti-155 positive								
	1	2	3	4	5	6	7	8	9
Onset age/Sex	35/M	52/M	16/M	19/M	55/F	50/M	35/M	15/M	20/M
Onset	A	C	A	A	C	C	C	C	C
Disease duration(month)	8	24	5	5	6	7	9	3	12
Clinical features									
Limb weakness	+	+	+	+	+	+	+	+	–
Sensory dysfunction	+	+	+	+	+	+	+	+	–
Ataxia	+	+	+	+	+	+	+	+	–
Cranial nerve involvement	–	–	Facial nerve	–	–	–	–	–	–
Tremor	–	+	–	–	–	–	+	–	–
CSF protein level (g/L)	2.65	3.85	3.52	3.40	1.14	2.27	3.41	3.37	0.31
Leucocyte (<5/µl)	4	2	8	2	6	4	2	8	2
Peripheral nerve MRI	NA	NA	(+)	NA	NA	NA	NA	NA	NA
Electrophysiological study									
Demyelination	+	+	+	+	+	+	+	+	+
Axonal damage	+	+	+	+	+	+	+	+	+
Reduced CV	+	+	+	+	+	+	+	+	+
Prolonged DML	+	+	+	+	+	+	+	+	+
CB	–	–	–	–	+	–	–	–	–
TD	–	–	–	–	+	–	–	–	–
Reduced CMAP amplitude	+	+	+	+	+	+	+	+	+
Treatment response									
Corticosteroids	+	+	NA	+	+	+	+	NA	NA
IVIG	NA	NA	–	NA	NA	NA	NA	NA	NA
PE	NA	NA	+	+	NA	NA	NA	+	NA
RTX	NA	NA	+	NA	NA	NA	NA	NA	NA
Cyclophosphamide	NA	+	NA	NA	NA	NA	NA	NA	NA

M, male; F, female; A, acute; C, chronic; CSF, cerebrospinal fluid; MRI, Magnetic resonance imaging; CV, conduction velocity; DML, distal motor latency; CB, conduction block; TD, temporal dispersion; CMAP, compound muscle action potential; IVIg, intravenous immunoglobulins; PE, plasma exchange; RTX, Rituximab; NA, not applicable.

In clinical characteristics and electrophysiological studies, the symbol "-" indicates negative or absent, and the symbol "+" indicates positive or present; in response to treatment, the symbol "-" indicates no response, and the symbol "+" indicates a response.

## 4 Discussion

The anti-NF155 antibody is the most prevalent antibody in AN. Previous studies have reported that patients with AN with anti-NF155 antibodies present with a specific clinical phenotype that is mainly characterized by young onset age, acute or subacute disease onset, limb weakness, sensory deficiency, ataxia, tremor, cranial nerve involvement, and significantly elevated CSF protein levels, and poor response to IVIg treatment (4, 5). The clinical characteristics of patients with AN with anti-NF155 antibodies in this study differed from those of previously reported patients, mainly in terms of electrophysiologic axonal damage. The obvious absence of tremor, pain, or cranial nerve involvement may suggest that the disease may have a broad spectrum of phenotypic variations and heterogeneity.

We compared data from nine patients with AN with anti-NF155 antibodies with those from 28 serologically negative patients with CIDP. The former group featured a younger age and onset age, more frequent ataxia, higher CSF protein levels, and more frequent axonal damage on electrophysiological examination. We did not find any other significant differences between the two groups of patients.

In our study, the mean onset age was 33 years in patients with AN and 50 years in the serologically negative patients with CIDP. Zhang et al. found mean onset age of 33 and 47 years, respectively, similar to our findings (7). The mean age at onset in the Japanese patients with CIDP reported by Devaux et al. was 31 and 48 years, respectively, and another study of Japanese patients with CIDP yielded similar results (6, 8). By comparison, two studies from Europe reported that the age at onset of patients with AN with anti-NF155 antibodies was younger than that of serologically negative patients with CIDP, at 40 and 52, respectively (9, 10). Besides, Delmont et al. prospectively tested sera from patients with suspected CIDP from France, Belgium, and Switzerland and found that the mean age of 15 patients with AN with NF155-IgG4 antibodies was younger than that of serologically negative patients with CIDP, with ages of onset of 54 and 66 years, respectively (11). This suggests that younger age and age at onset may be more representative of Asian populations, possibly due to ethnic differences; however, larger sample sizes are needed for further validation.

Ataxia is one of the clinical features that distinguishes patients with AN from serologically negative patients with CIDP, and our findings support this conclusion (6). Studies appear to be lacking on the specific mechanisms by which ataxia occurs in patients with CIDP with anti-NF155 antibodies. We hypothesize that ataxia may result from demyelination of sensory neurons or other unexplored mechanisms.

**Figure 2:**
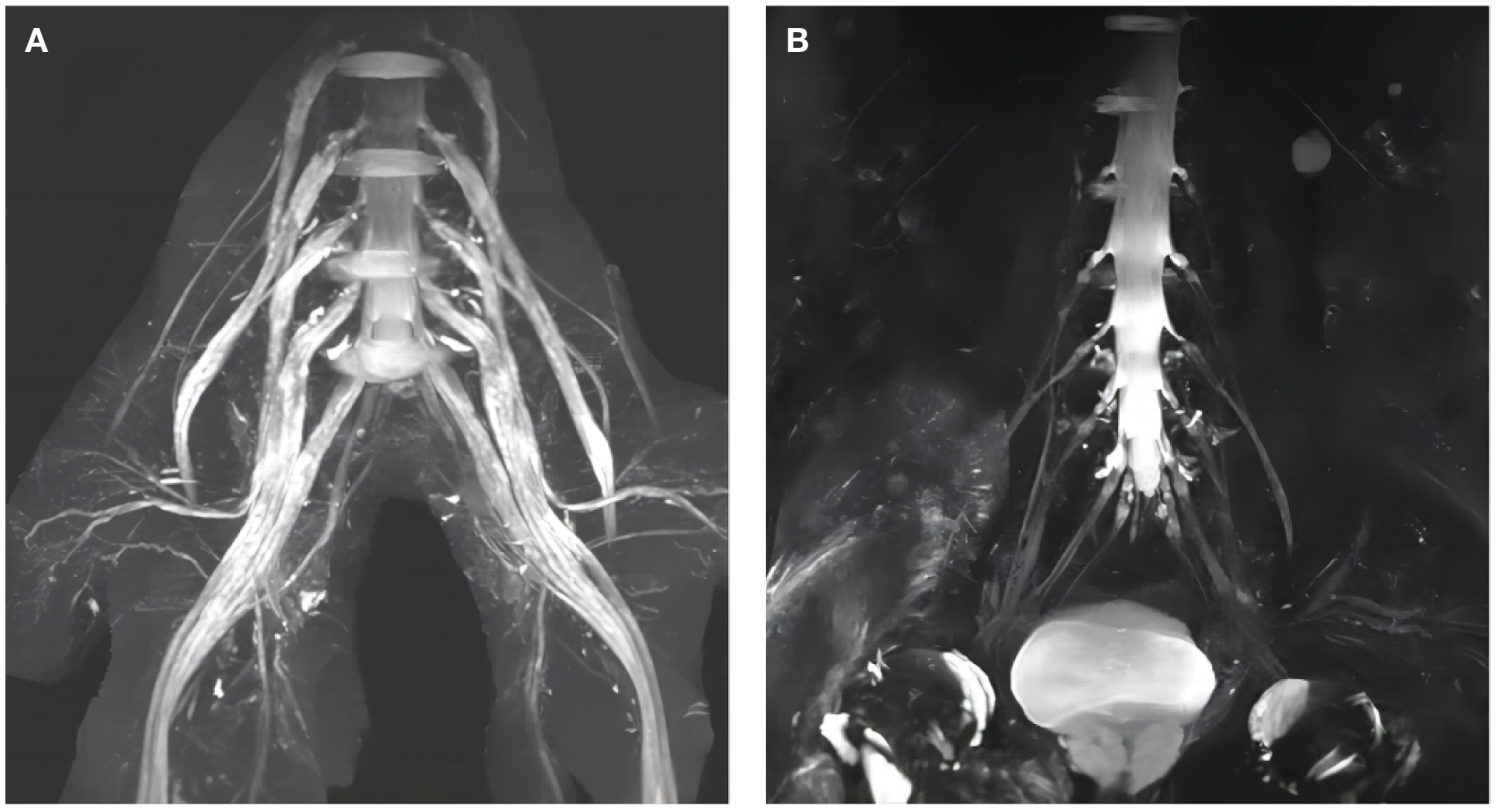
Lumbosacral plexus MRI findings. **(A)** Diffuse thickening of the lumbosacral nerve roots of a patient with AN with anti-NF155 antibodies; **(B)** Normal lumbosacral nerve roots of a serologically negative patient with CIDP.

CSF albuminocytologic dissociation is a common phenomenon in CIDP, and the vast majority of patients with AN with anti-NF155 antibodies had markedly elevated CSF protein levels−up to more than 3.8 g/L−which were much higher than those in serologically negative patients with CIDP. At the meantime, magnetic resonance imaging (MRI) of peripheral nerves showed thickening of nerve roots in the cervical plexus or lumbosacral plexus, which is similar to what has been reported in previous studies (8, 12) (**Figure 2**). The reason for the elevated CSF protein levels remains unclear and may be related to thickening of the nerve root. Previous studies have found significant involvement of nerve roots of peripheral nerve in patients with AN compared to serologically negative patients with CIDP (6). Because of the absence of pathological manifestations of repeated demyelination and myelin regeneration in patients with AN as well as the lack of histological examination of proximal nerve roots, the mechanism of nerve roots thickening in patients with AN remains to be further explored. This antibodies may be involved in the elevation of CSF protein levels and thickening of nerve roots by some complex mechanism.

Electrophysiological examination revealed frequent involvement of the motor and sensory nerves in patients with AN with anti-NF155 antibodies, and all patients had axonal damage, with demyelination predominating in the vast majority of the patients. This may correlate with the localization of NF155 in NR and its close relationship with axon, perhaps explained by the fact that nerve conduction depends on the structural and functional integrity of myelinated nerve fibers. NR consists of the node, paranode, and juxta-paranode, structurally and functionally specialized regions on the axon of myelinated nerve fibers that ensure the “jumping” conduction of nerve impulses on myelinated fibers (13). The structural basis is “septate-like” junctions at the paranodes, consisting of NF155 located on myelin loops and CNTN1 and Caspr1 on the axolemma, which tightly bind the myelin sheath to the axon and act as electrical and molecular barriers limiting diffusion of ions and ion channels (14). Autoantibodies attacking on NF155 injure septate-like junctions leading to detachment of the myelin loops from the axon, thus affecting peripheral nerve conduction (15, 16). Mutant animal models have demonstrated impaired septal-like junction formation in the paranodal region and decreased peripheral nerve conduction velocity in mice lacking NF155, CNTN1, or Caspr1 (11, 17). All patients with AN in this study showed decreased or even inability to elicit CMAP amplitude, and the electromyography results of eight patients suggested neurogenic changes in the tested muscles, which suggests axonal damage to the peripheral nerves. Previous pathological findings did suggest that patients with AN with anti-NF155 antibodies have impaired formation of septate-like junctions, leading to secondary axonal damage (18). It can be hypothesized that NF155 may be a target antigen for autoantibody-mediated axonal damage in patients with AN with anti-NF155 antibodies.

Corticosteroids, IVIg, and plasma exchange therapy are first-line treatment options for CIDP, with most patients responding favorably. In this study, most patients with AN with anti-NF155 antibodies who received immunotherapy experienced varying degrees of improvement in their symptoms. However, the therapeutic efficacy of IVIg was unsatisfactory, which is in line with previous studies (19, 20). The poor response to IVIg may be explained by the fact that the predominant subtype of the anti-NF155 antibody is IgG4, which lacks complement-activating capability and has a low affinity for Fc-receptors, which constitute the mechanism of action of IVIg (6). Thus, antibody nature determines the lack of response to IVIg. Other patients with predominantly IgG1, IgG2, or IgG3 antibodies early in the course of the disease, responded favorably to IVIg; when the disease progressed to the middle and late stages, the antibody subtype shifted to IgG4, which may account for the different responses to IVIg at different stages of the disease (21, 22). Therefore, it is important to perform antibody typing and early treatment. Since there was only one patient with AN treated with IVIg in this study, further confirmation regarding the effectiveness of IVIg is still needed from studies with larger sample sizes.

Notably, we found no significant difference in the efficacy of corticosteroids between the two groups. We conjectured that the response to corticosteroids in the two groups may be independent of the serological status of the antibodies. Rituximab (RTX) is an option for patients with AN who do not respond well to first-line therapy (23). However, one patient with AN with anti-NF155 antibodies was treated with RTX and, despite symptomatic relief, the drug was discontinued because of oliguria and bilateral lower-extremity edema. Long-term observations are still needed to evaluate the effects of RTX treatment. Overall, the vast majority of patients with AN patients in this study responded well to immunotherapy.

This study has some limitations. First, the number of patients with CIDP with anti-NF155 antibodies was small. Second, the antibody subtypes were unknown, preventing a more detailed characterization of the features corresponding to each subtype. Finally, complete and detailed follow-up information on efficacy could not be obtained because this was a retrospective study. Large-sample, multicenter prospective studies and long-term follow-up studies are needed.

In summary, the analysis and comparison revealed patients with AN with anti-NF155 antibodies have unique clinical characteristics that differ from serologically negative patients with CIDP, such as younger age and onset age, more frequent ataxia, significantly higher CSF protein levels, and more frequent electrophysiological manifestations of axon damage. Admittedly, these antibody-positive neuropathies should not be categorized as CIDP but rather as a new disease entity different from CIDP. If a patient presents with young onset, limb weakness, ataxia, markedly high CSF protein levels, and poor response to IVIg, detection of nodal or paranodal antibodies is recommended to help in the early diagnosis and guide treatment of AN.

## Data availability statement

The raw data supporting the conclusions of this article will be made available by the authors, without undue reservation.

## Ethics statement

The studies involving humans were approved by the Ethics Committee of the First Affiliated Hospital of Zhengzhou University. The studies were conducted in accordance with the local legislation and institutional requirements. Written informed consent for participation was not required from the participants or the participants’ legal guardians/next of kin in accordance with the national legislation and institutional requirements.

## Author contributions

XH: Writing – review & editing, Writing – original draft, Data curation. LW: Writing – review & editing. JL: Writing – review & editing. SL: Writing – review & editing. YG: Writing – review & editing. JZ: Writing – review & editing, Supervision, Conceptualization. HL: Writing – review & editing. YJ: Resources, Writing – review & editing.
