# Supplementary material for: Clinical characteristics of patients with autoimmune nodopathy with anti-neurofascin155 antibodies
Source: Front Immunol. 2024 Apr 25;15:1345953. doi: 10.3389/fimmu.2024.1345953 (PMC11079118; doi:10.3389/fimmu.2024.1345953)
Supplement: Supplementary file 1 [file Table_1.docx]

**Supplementary table 1.** Electrophysiology in nine patients with AN.

| EMG | | Number of cases | | | | | | | | |
| --- | --- | --- | --- | --- | --- | --- | --- | --- | --- | --- |
|  |  | 1 | 2 | 3 | 4 | 5 | 6 | 7 | 8 | 9 |
| Motion conduction |  |  |  |  |  |  |  |  |  |  |
| Median nerve. L | DML (ms) | 10.5 | 9.5 | 7.5 | 13.6 | 7.2 | 7.1 | 8.6 | 5.4 | 4.6 |
|  | MCV (m/s) | 34 | 20 | 27.3 | 21 | 61.1 | 20.7 | 17 | 54 | 34 |
|  | CMAP (mV) | 9.6 | 7.1 | 3.3 | 0.6 | 3.8 | 4.3 | 2.0 | 9.3 | 6.4 |
|  | F-wave Latency (m/s) | ND | 74.1 | - | 23.2 | 38.8 | ND | ND | 39.6 | ND |
| Median nerve. R | DML (ms) | 10.3 | 11.7 | 8.1 | 13.6 | 6.4 | 8.0 | 8.6 | 6.1 | 6.3 |
|  | MCV (m/s) | 27 | 23 | 28.1 | 11 | 43.6 | 22 | 15 | 54 | 35 |
|  | CMAP (mV) | 7.4 | 0.4 | 2.5 | 0.2 | 5.0 | 1.6 | 4.3 | 11.7 | 1.2 |
|  | F-wave Latency (m/s) | ND | 62.2 | - | 26.0 | 37.7 | ND | ND | 38.0 | ND |
| Ulnar nerve. L | DML (ms) | ND | 6.0 | ND | 10.4 | 6.2 | 5.4 | ND | 5.2 | 3.5 |
|  | MCV (m/s) | ND | 22 | ND | 16 | 58.2 | 25.5 | ND | 50 | 28 |
|  | CMAP (mV) | ND | 6.7 | ND | 0.5 | 3.2 | 4.3 | ND | 6.8 | 10.5 |
|  | F-wave Latency (m/s) | ND | ND | ND | ND | ND | 65.3 | ND | ND | ND |
| Ulnar nerve. R | DML (ms) | ND | 6.5 | ND | 10.6 | 4.0 | 5.1 | ND | 5.3 | 4.0 |
|  | MCV (m/s) | ND | 20 | ND | 15 | 47.1 | 23.7 | ND | 53 | 32 |
|  | CMAP (mV) | ND | 2.0 | ND | 1.5 | 3.6 | 5.2 | ND | 8.3 | 4.8 |
|  | F-wave Latency (m/s) | ND | ND | ND | ND | ND | 66.8 | ND | ND | ND |
| Peroneal nerve. L | DML (ms) | - | - | - | - | - | - | 14.9 | 9.6 | 9.2 |
|  | MCV (m/s) | - | - | - | - | - | - | 23 | 43 | 31 |
|  | CMAP (mV) | - | - | - | - | - | - | 0.3 | 1.3 | 0.4 |
|  | F-wave Latency (m/s) | ND | ND | ND | ND | ND | ND | ND | ND | ND |
| Peroneal nerve. R | DML (ms) | - | - | - | - | 6.8 | - | - | 10.8 | 7.1 |
|  | MCV (m/s) | - | - | - | - | 36.7 | - | - | 34 | 34 |
|  | CMAP (mV) | - | - | - | - | 0.8 | - | - | 0.5 | 2.8 |
|  | F-wave Latency (m/s) | ND | ND | ND | ND | ND | ND | ND | ND | ND |
| Tibial nerve. L | DML (ms) | - | - | 14.3 | - | 10.9 | - | - | 11.2 | 5.7 |
|  | MCV (m/s) | - | - | 18.6 | - | 34.2 | - | - | 49 | 33 |
|  | CMAP (mV) | - | - | 0.04 | - | 0.4 | - | - | 6.1 | 2.1 |
|  | F-wave Latency (m/s) | ND | - | ND | ND | ND | ND | ND | 90.8 | ND |
| Tibial nerve. R | DML (ms) | - | - | 20.5 | - | 10.8 | - | - | 9.5 | 5.8 |
|  | MCV (m/s) | - | - | 25.5 | - | 36.9 | - | - | 43 | 30 |
|  | CMAP (mV) | - | - | 0.03 | - | 0.4 | - | - | 5.8 | 2.0 |
|  | F-wave Latency (m/s) | ND | - | ND | ND | ND | ND | ND | 78.8 | ND |
| Sensory conduction |  |  |  |  |  |  |  |  |  |  |
| Median nerve. L | SNAP (μV) | 5 | - | - | - | - | - | - | 6 | - |
|  | SCV (m/s) | - | - | - | - | - | - | - | 45 | - |
| Median nerve. R | SNAP (μV) | 2 | - | - | - | - | - | - | - | - |
|  | SCV (m/s) | - | - | - | - | - | - | - | - | - |
| Ulnar nerve. L | SNAP (μV) | ND | - | ND | - | - | - | ND | 5 | - |
|  | SCV (m/s) | ND | - | ND | - | - | - | ND | 37 | - |
| Ulnar nerve. R | SNAP (μV) | ND | - | ND | - | - | - | ND | 5 | - |
|  | SCV (m/s) | ND | - | ND | - | - | - | ND | 43 | - |
| Superficial peroneal nerve. L | SNAP (μV) | ND | ND | ND | ND | - | - | ND | ND | ND |
|  | SCV (m/s) | ND | ND | ND | ND | - | - | ND | ND | ND |
| Superficial peroneal nerve. R | SNAP (μV) | ND | ND | ND | ND | - | - | ND | ND | ND |
|  | SCV (m/s) | ND | ND | ND | ND | - | - | ND | ND | ND |
| Sural nerve. L | SNAP (μV) | - | - | - | - | - | - | - | 15 | - |
|  | SCV (m/s) | - | - | - | - | - | - | - | 45 | - |
| Sural nerve. R | SNAP (μV) | - | - | - | - | - | - | - | 22 | - |
|  | SCV (m/s) | - | - | - | - | - | - | - | 45 | - |

DML, distal motor latency; MNCV, motor nerve conduction velocity; CMAP, compound muscle action potential; SNAP, sensory nerve action potential; SNCV, sensory nerve conduction velocity; L, left; R, right; “-” indicated not evoked; ND, not done.
